# Metabolic Flexibility and Innate Immunity in Renal Ischemia Reperfusion Injury: The Fine Balance Between Adaptive Repair and Tissue Degeneration

**DOI:** 10.3389/fimmu.2020.01346

**Published:** 2020-07-07

**Authors:** Alessandra Tammaro, Jesper Kers, Angelique M. L. Scantlebery, Sandrine Florquin

**Affiliations:** ^1^Department of Pathology, Amsterdam UMC, Amsterdam Infection & Immunity Institute, University of Amsterdam, Amsterdam, Netherlands; ^2^Department of Pathology, Leiden University Medical Center, Leiden, Netherlands; ^3^Biomolecular Systems Analytics, Van ‘t Hoff Institute for Molecular Sciences (HIMS), University of Amsterdam, Amsterdam, Netherlands

**Keywords:** kidney transplantation, tubular repair, innate immunity, cell death, mitochondria, senescence

## Abstract

Renal ischemia reperfusion injury (IRI), a common event after renal transplantation, causes acute kidney injury (AKI), increases the risk of delayed graft function (DGF), primes the donor kidney for rejection, and contributes to the long-term risk of graft loss. In the last decade, epidemiological studies have linked even mild episodes of AKI to chronic kidney disease (CKD) progression, and innate immunity seems to play a crucial role. The ischemic insult triggers an acute inflammatory reaction that is elicited by Pattern Recognition Receptors (PRRs), expressed on both infiltrating immune cells as well as tubular epithelial cells (TECs). Among the PRRs, Toll-like receptors (TLRs), their synergistic receptors, Nod-like receptors (NLRs), and the inflammasomes, play a pivotal role in shaping inflammation and TEC repair, in response to renal IRI. These receptors represent promising targets to modulate the extent of inflammation, but also function as gatekeepers of tissue repair, protecting against AKI-to-CKD progression. Despite the important considerations on timely use of therapeutics, in the context of IRI, treatment options are limited by a lack of understanding of the intra- and intercellular mechanisms associated with the activation of innate immune receptors and their impact on adaptive tubular repair. Accumulating evidence suggests that TEC-associated innate immunity shapes the tubular response to stress through the regulation of immunometabolism. Engagement of innate immune receptors provides TECs with the metabolic flexibility necessary for their plasticity during injury and repair. This could significantly affect pathogenic processes within TECs, such as cell death, mitochondrial damage, senescence, and pro-fibrotic cytokine secretion, well-known to exacerbate inflammation and fibrosis. This article provides an overview of the past 5 years of research on the role of innate immunity in experimental and human IRI, with a focus on the cascade of events activated by hypoxic damage in TECs: from programmed cell death (PCD) and mitochondrial dysfunction-mediated metabolic rewiring of TECs to maladaptive repair and progression to fibrosis. Finally, we will discuss the important crosstalk between metabolism and innate immunity observed in TECs and their therapeutic potential in both experimental and clinical research.

## Introduction

Kidney diseases are a growing health problem, considered to have a direct and indirect impact on morbidity and mortality worldwide, by increasing the risks associated with the major killers, such as cardiovascular diseases, diabetes, hypertension, and infection (1). The timely identification and management of kidney diseases represent the most effective strategy to sustainably address the growing global burden and prevent the progression to end-stage renal disease (ESRD).

In 2010, 2.62 million people received dialysis worldwide, and the need for dialysis was projected to double by 2030. Despite being a life-saving treatment, dialysis is merely a supportive measure. Indeed, the life expectancy and the quality of life in dialysis patients is much lower compared to the general population (2). Basic research studies have identified many targets to delay the progression of kidney disease; however, only few of these promising results can be recapitulated in clinical studies, and yet do not represent a great alternative to dialysis or transplantation.

The life-sustaining job of the kidneys comprises filtering and reabsorbing about 180 liters of fluid from the bloodstream every 24 h. As a consequence, it is the organ with the highest metabolic rate as determined by tubular epithelial cell (TEC) metabolism (3). TECs, the most abundant cell type in the kidney, are densely packed with mitochondria. These cells combust fatty acids to generate adenosine triphosphate (ATP) through oxidative phosphorylation (4). Proper mitochondrial function and metabolism of these cells is crucial for the high transport and reabsorption activities. Given the high metabolic demand, the kidneys are sensitive to decreased blood oxygenation and perfusion. Whenever the kidneys experience extremely low oxygen exposure, or metabolic substrates become inadequate, acute kidney injury (AKI) occurs.

AKI is defined as an abrupt reduction in kidney function that results in disturbances in the milieu intérieur and the retention of uremic toxins that especially influence the cardiovascular, immune, and nervous system. With an incidence of 9–15% of hospital admissions and up to 40% in critically ill patients, AKI is a major cause of morbidity, mortality (40–70% for the critically ill needing dialysis), increased health-care costs, and chronic kidney disease (CKD) (5, 6). Although research has made great efforts to understand the pathogenesis of AKI, the only available therapy is still supportive (dialysis) and has not changed for decades. Additionally, AKI patients have a greater risk to develop chronic complications later in life (7). A systematic review and meta-analysis comprising estimates from more than 2,000,000 individuals identified AKI as a risk factor for new or progressive CKD (hazard ratio [HR] = 2.67), ESRD (HR = 4.81), and mortality (HR = 1.80) (7). These important clinical findings have resulted in a shift toward studies that investigate the link between experimental AKI and progressive kidney fibrosis and failure. Pattern recognition receptors (PRRs), the sensors of the innate immune system, are one of the major players determining short-term outcomes after experimental AKI, a topic of many good reviews (8, 9). In this review, we will discuss recent data showing that danger signals and PRRs are involved in cell fate decisions, metabolism, and mitochondrial function in TECs, thereby determining not only whether a pro- or anti-inflammatory phenotype will emerge, but also the success of regeneration and repair. We specifically intend to highlight the link between metabolism and innate immunity in TECs and the altered cell phenotype that occurs during experimental and human ischemia reperfusion injury (IRI)-induced AKI.

## AKI and Experimental Ischemia-Reperfusion Injury

The pathophysiology of AKI is complex. One of the major causes of AKI is IRI (10, 11). IRI is an inevitable event during renal transplantation and is responsible for delayed graft function (DGF), resulting in loss of vital kidney parenchyma and priming of adaptive immune responses that initiate rejection, which altogether lead to graft loss (10, 12). DGF is a clinical syndrome defined as the need for renal replacement therapy in the early phase after transplantation in order to support the function of the newly acquired renal transplant (12). Besides renal transplantation, IRI can also develop in the context of other diseases with low perfusion and/or oxygenation states, including thrombotic diseases, sepsis, trauma, and cardiac surgery (13). Experimental IRI in the mouse is the most widely used preclinical model to mimic human AKI. Despite having many limitations, particularly regarding the immune response (14) it is still the most valuable tool for understanding AKI pathophysiology.

### The Early Phase of Experimental IRI Is Characterized by Programmed Cell Death That Activates a Pro-inflammatory Innate Immune Response

In experimental IRI, following the hypoxic event, TECs, especially the proximal TECs located in the S3 segment of the nephron at the cortico-medullary area, are unable to maintain adequate intracellular ATP levels for the essential processes mentioned earlier. Additionally, restoration of blood perfusion, followed by re-oxygenation of the kidney, provokes the production of reactive oxygen species (ROS), eliciting mitochondrial dysfunction. This, together with the ATP-depletion, leads to cell activation and injury, and if severe enough, can lead to programmed cell death (PCD) and secondary necrosis, the hallmark of early IRI (15).

PCD is essential, not only for the maintenance of cellular homeostasis but also in response to irreparable damage caused by injury or disease (15–17). The pathways of cell death execution can present as a diverse morphological pallet in the spectrum from apoptotic to necrotic, with a corresponding degree of danger-associated molecular pattern (DAMP) release and inflammatory potential (18–20). DAMPs are a heterogeneous group of ligands, which are constitutively expressed in different biological compartments of cells hidden from the innate immune system. DAMPs are recognized by a broad spectrum of PRRs, in order to elicit an innate inflammatory response (19).

It has been observed that the severity of the injury determines the path by which cell death is realized, indicating a possible role for PRRs in guiding cell fate decisions by integrating signals from the microenvironment (21). Various currently recognized forms of PCD (18, 22), including necroptosis, pyroptosis, ferroptosis, mitochondrial permeabilization transition (MPT)-mediated regulated necrosis, and parthanatos are initiated as a response to hypoxic injury, either directly or indirectly, and blocking their crucial pathways during the early phase of IRI generally leads to reduced necrotic (tubular) damage, reduced inflammation, preservation of renal function, and reduced mortality (15). PRRs are known inducers of PCD, particularly necroptosis, pyroptosis, and apoptosis, which is why these modalities are discussed in more detail below.

### Pattern Recognition Receptors Are Involved in Cell Death Signaling During IRI

In necroptosis, receptor-interacting protein (RIP) kinase-1 and 3 interaction via their RIP–homotypic interacting motif (RHIM) (23) leads to phosphorylation and activation of RIPK3. In turn, RIPK3 catalyzes the phosphorylation and oligomerization of mixed lineage kinase domain–like (MLKL) (24), thereby inducing a molecular switch that leads to plasma membrane rupture and cell death (23–26). Blocking components of the necroptosis pathway in a similar renal phenotype of reduced tubular necrosis, reduced inflammation, better preservation of renal function, and reduced mortality in the first period after reperfusion (27). A recent report by Chen and colleagues showed, in a chimeric bone marrow transplantation model, that in the early phase of IRI, RIPK3 and MLKL in kidney parenchymal cells (including proximal TECs, as shown by *in vitro* studies) are important for initiation of the vicious inflammatory circle, but that pyroptosis in macrophages is more important in the later stage after reperfusion, suggesting temporal variation in cell death modalities during the course of IRI (28). Pyroptosis is a necrotic form of cell death most often observed in immune cells, such as macrophages and dendritic cells (DCs) (18). During pyroptosis, the presence of DAMPs initiates inflammasome formation, which activates both caspase-1 and caspase-11 (29–31). An effector function of these caspases is to process the inactive precursors of IL-18 and IL-1beta, leading to an intracellular accumulation of pro-inflammatory cytokines (31). These caspases also induce plasma membrane rupture, and essentially cell death, through the cleavage of gasdermin D (GSDMD) (32). The inevitable release of IL-18 and IL-1beta makes this form of cell death highly inflammatory (33). There is some debate as to whether pyroptosis occurs in renal cells as well, however, Yang et al. suggest the occurrence of pyroptosis in TECs based on a significant increase in pyroptosis-related proteins following IRI (34). A recent report by Miao et al. suggests the direct involvement of pyroptosis in IRI and cisplatin toxicity based on *Casp11* KO mice (35). In additional experiments they showed that in *Gsdmd* KO mice, renal tubular damage was less severe, and urinary IL-18 levels were reduced upon cisplatin toxicity (35). Although very suggestive, we do not know whether *Gsdmd* KO mice have the same phenotype in IRI compared to cisplatin toxicitiy *in vivo*. Apoptosis is considered a more quiescent form of regulated cell death due to the swift clearance of apoptotic bodies by phagocytes. Apoptosis is initiated through an intrinsic route via mitochondrial outer membrane permeabilization (MOMP) or an extrinsic route via death or dependence receptors (36, 37). Death receptors can initiate apoptosis via cognate ligand binding (e.g., FAS or TNFR1 signaling) whereas dependence receptors initiate apoptosis when there is a lack of ligand binding (i.e., reduced homeostatic survival signaling via e.g., the netrin 1 receptors) (38). After initiation, executioner caspases-3 and−7 are responsible for neat cellular and nuclear fragmentation during apoptosis, releasing “find-me” signals, and flagging apoptotic bodies to be phagocytosed via “eat-me” signals (39). Apoptosis is believed to play a minor role in the early pro-inflammatory phase after reperfusion. Multiple reports have shown that pharmacologically inhibited apoptosis by a pan-caspase inhibitor zVAD or genetic KO of executioner caspase-3 did not reduce but rather exaggerated renal tubular damage and failure (40, 41). In the long-term, *Casp3* KO mice appeared to have less peritubular capillary rarefaction, less activated interstitial fibroblasts, less interstitial fibrosis, and evidence of less tubular hypoxia after reperfusion, suggesting a potentially interesting link between late peritubular capillary apoptosis and endothelial-mesenchymal transition and/or pericyte-fibroblast transdifferentiation (41). PRRs can initiate regulated cell death in multiple ways. Toll-like receptor (TLR) signaling via MYD88 results in activation of NFkB, transcriptionally regulating multiple cytokines that can subsequently induce regulated cell death via para- and autocrine signaling to death receptors. However, a more direct route of cell death initiation by TLRs is via Toll/IL-1R domain-containing adaptor-inducing interferon (IFN)-beta (TRIF). TRIF can initiate apoptosis via FADD- and caspase-8-dependent pathways. TRIF also contains a RHIM domain, and could therefore function as a docking site for the RIPK3-MLKL complex during necroptosis initiation (42), as was shown for TLR3 (43). TLR-TRIF-induced active caspase-8 was able to cleave Gasdermin D in macrophages inducing pyroptosis (44), suggesting the bypassing of the inflammasome in these cells.

## (mal)Adaptive Repair Responses as a Model for AKI-to-CKD Progression

Tubular regeneration and successful renal repair after an episode of AKI can be observed in the majority of surviving patients, especially in cases of mild injury (45–47). Adaptive tubular repair depends on the presence of an appropriate microenvironment, in which inflammation and tubular response to damage are balanced. In the adaptive repair, surviving TECs undergo dedifferentiation and proliferation in order to restore a functional epithelium. However, in case of severe or repetitive injuries or aged kidneys, maladaptive repair of proximal tubules can occur, which can contribute to progressive renal fibrosis (47). Maladaptive repair of kidney tissue after AKI is characterized by rarefaction of peritubular capillaries, interstitial fibrosis and tubular atrophy, glomerulosclerosis, and vascular remodeling, which interfere with repair and eventually lead to a decline in renal function. Therefore, AKI-to-CKD should be regarded as accelerated renal aging (47, 48). As the determinants of renal aging and CKD overlap (49), identifying patients with premature renal aging could be a strategy to identify AKI survivors at risk for CKD.

Among the culprits in the AKI-to-CKD progression, is the persistence of a senescent state in TECs (45). Senescence describes a proliferative arrest with changes in chromatin organization, gene transcription, and protein secretion, which can occur as a response to cell stress and aging (50). Given the high degree of pro-inflammatory molecules released by senescent TECs, it remains elusive whether progressive accumulation of senescent TECs is causally related to an aberrant innate immune response. Recent results from our group and others (51–53) point toward a role for TEC-mediated inflammation, innate immunity, and mitochondrial metabolism in senescence and fibrosis. Given that these mechanisms fall into the new discipline of immunometabolism, and TECs can be regarded as part of the renal innate immune system, further studies are required to characterize the role of immunometabolism in AKI-to-CKD progression.

### Innate Immune Receptors as Gatekeeper of Damage and Repair

The role of the innate immune system was originally to combat infections. However, we now know that its role extends beyond that to include the surveillance of tissue homeostasis, by detecting distinct DAMPs released during tissue injury. Our group pioneered the discovery of TEC-associated innate immune sensors having a crucial role in the initiation of the injury response during IRI, shedding a novel light on the role of TECs as innate immune cells of the kidney (54). Experimental data suggests that the DAMPs released by necrotic cells activate the inflammatory signal initiated through TLRs, and their synergistic receptors, the Nod-like receptors (NLRs) and the NLRP3 inflammasome (55, 56). Initial considerations suggested that pharmaceutical intervention to block TEC-induced innate immune cell signaling could lead to novel therapeutics against renal tissue inflammation and injury after IRI (54, 57). However, in the last decade, seminal studies on the role of innate immune sensors and their ligands in renal IRI have provided an additional prospective: the innate immune sensors translate kidney injury into an immune response, essential for shaping adaptive tubular repair and kidney regeneration. Thus, the tubular innate immune response to IRI seems to be a very well-orchestrated phenomenon (58). After the early inflammatory phase, macrophage populations assume a reparative phenotype that is characterized by the production of numerous growth factors, including Platelet-derived growth factor (PDGF), Transforming growth factor beta 1 (TGF-beta1), Insulin-like Growth Factor I (IGF-1), and Vascular endothelial growth factor A (VEGF-A) that promote tubular regeneration (59–61). Long-term sustained inflammation is detrimental, but the absence of the inflammatory response can also predispose to the development and progression of CKD (8). Whether this is dependent on a faulty inflammatory response or the inability of TECs to regenerate due to extensive damage is still not completely understood. Improving our understanding of the role of innate immune receptors, not only in the early pro-inflammatory phase during IRI, but also in (mal)adaptive tubular repair, is therefore crucial for the development of specific therapies or prevention of AKI and its detrimental sequelae.

### Mitochondrial Dysfunction and Metabolic Reprogramming

Mitochondrial injury, fragmentation, and ROS generation induce aberrant tubular inflammation and are central mediators of AKI, as well as the AKI-to-CKD progression (62–66). Oxygen tension in TECs is crucial for proper mitochondrial function. When this becomes inadequate, mitochondrial respiration is inhibited and the kidney undergoes a metabolic rewiring toward glycolysis, thereby decreasing ATP production (67). Mitochondrial number and integrity (through fission and fusion processes) (65, 66) are associated with accelerated tubular repair and improved survival after IRI, suggesting that in order to achieve adaptive tubular repair, restoration of mitochondrial homeostasis is an indispensable event (64). Thus, metabolic flexibility seems to be a crucial ability of the tubular epithelium to quickly adapt to the hypoxic environment. However, in order to promote repair, TECs should be able to return to their primary fuel source, and when this flexibility is impaired and metabolic rewiring persists during repair, this leads to a failed re-differentiation and mesenchymal arrest (68).

Hypoxia is likely not the only cause of reduced oxygen availability in the kidney. The widening of interstitial spaces by edema and inflammation and the regression of capillaries during fibrosis could also be involved. Indeed, pathologic hypoxia persists as fibrosis develops and could thus prevent epithelial recovery through feedback effects, ultimately leading to tubular atrophy (69). Consequent to hypoxia, tubular repair after AKI could be impaired by oxidative stress and growth arrest or senescence, which represents the known adverse effects of hypoxia (51, 52, 70, 71). TECs rely mostly on fatty acid oxidation (FAO) for their functions (72), which requires mitochondria and oxygen for efficient ATP generation. All the enzymes required for FAO reside in the mitochondrial matrix, and proper functioning of the mitochondrial cristae is necessary to provide substrates for the respiratory complex. Recently, several groups have reported that mitochondrial function and energy metabolism are involved in the progression from AKI-to-CKD.

Within mitochondria, the coenzyme Nicotinamide adenine dinucleotide (NAD^+^) carries high-energy electrons from FAO to the electron transport chain. NAD^+^ is, therefore, a rate-limiting catalyst for FAO (67). In other terms, decreased NAD^+^ availability results in impaired energy metabolism in these cells (73). Hypoxia and aging (74, 75) have been known to induce NAD^+^-consuming enzymes, which lowers NAD^+^ availability. Tran and colleagues recently showed that renal tubular cell NAD^+^ levels are suppressed in IRI-induced AKI, and this reduction in NAD^+^ may impede FAO, reduce ATP generation, and elevate susceptibility to AKI stressors (76). They identified a novel function of the mitochondrial biogenesis regulator PPAR-gamma-coactivator-1alpha (PGC1-alpha) to induce the enzymes that sequentially convert the amino acid tryptophan to NAD^+^, after IRI. Tubular PGC1-alpha expression protects against hypoxia-related stress and enhancing NAD^+^ could effectively mimic PGC1-alpha's effects in the tubule (76). Interestingly, biopsies of human AKI showed reduced PGC1-alpha expression (77).

The same group, through a metabolomics study, has demonstrated the elevation of urinary quinolinic acid (uQuin) in murine AKI. Quin becomes NAD^+^ through the action of quinolinate phosphoribosyltransferase (QPRT) and subsequent enzymes. The elevation of uQuin suggested suppression of QPRT during AKI. Genetic targeting of *Qprt* showed enhanced susceptibility to IRI and recapitulated the majority of the urinary metabolic changes measured in experimental AKI (78). Additionally, few other studies described that either *de novo* NAD^+^ biosynthetic pathway activation (79) or replenishment by means of NAD^+^ precursors (80) are able to protect the kidney from ischemic damage. This is of great importance given that in mammals, only the kidney and the liver exhibit appreciable *de novo* NAD^+^ biosynthesis (79, 81). Taken together, these encouraging studies points toward a therapeutic potential of NAD^+^ enhancers in AKI and its long-term sequelae.

Apart from PGC1-alpha, the AMP-activated protein kinase (AMPK) is a promising component of a signaling cascade that may modulate the severity of ischemic injury (82). AMPK is a ubiquitously expressed serine-threonine kinase that serves as an important intracellular energy sensor. It is activated by conditions that deplete ATP and alter the AMP:ATP ratio, including ischemia and glucose deprivation. AMPK stimulates FAO, glucose uptake, and glycolysis, while downregulating ATP-utilizing systems. Other targets of AMPK include pathways modulating inflammation, apoptosis, angiogenesis, blood flow, and maintenance of cell polarity, with or without energetic stress (83). Metformin, a widely used drug for the treatment of type II diabetes mellitus, enhances AMPK activity (84). AMPK pre-activation partially ameliorates renal IRI *in vivo* but also long-term sequelae after IRI (84).

Defective FAO in TECs plays a pivotal role in renal aging (85) and fibrosis (72), but studies directly linking defective FAO to renal IRI are currently missing. The Susztak group showed that the dramatic repression of FAO is induced by TGF-beta signaling (72), which seems to play a role in the early events of renal IRI, therefore, it would be interesting to investigate whether failure to fully return to FAO fuel after IRI might be underlying the AKI-to-CKD transition, or whether defective FAO is associated with a senescent tubular phenotype. Although fatty acid accumulation has been shown to be associated with lipid deposition, this is not *per se* enough to drive fibrosis.

Interestingly, TGF-beta1 stimulates the Warburg-like metabolic reprogramming in kidney cells, which is relevant because it mirrors the metabolic state during AKI (72). Metabolic reprogramming, toward glycolysis, rapidly generates ATP and involves the enzyme Pyruvate Kinase M2 (PKM2), which is involved in the last step of glycolysis (86). Zhou and colleagues found that disabling PKM2 resulted in a significant increase in cell-repair and a concomitant decrease in energy generation, leading to significant protection against kidney injury in mice (87). A key molecule in this process is nitric oxide (NO), which can be transported to different proteins through Co-enzyme A, thereby, switching off their activity. PKM2 is one such protein. Indeed, adding NO to PKM2 activates repair, suggesting it as an important mechanism that can be used to determine whether kidney cells are using their pathways for energy or repair. The same team found that a protein called AKR1A1 could remove NO from PKM2, thereby switching it on and re-activating a robust energy-generating process. Disabling AKR1A1 protected the kidney from disease by stimulating repair (87).

**Figure 1:**
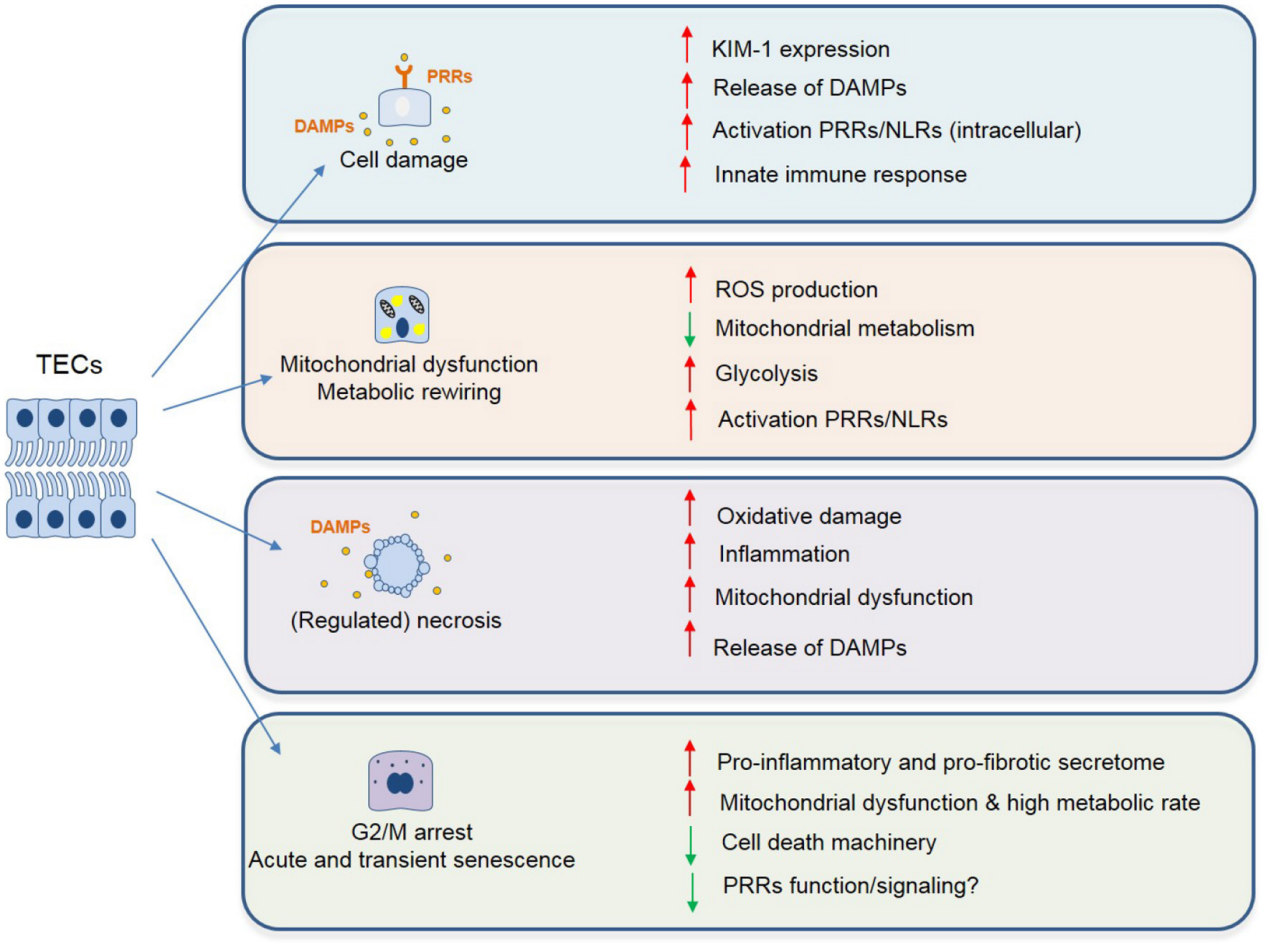
Phenotypic changes described in TECs following IRI associated with maladaptive tubular repair and progressive renal interstitial fibrosis. These changes include but are not limited to: cell damage (chronic inflammation with persistent cytokine production and immune cell infiltrate), mitochondrial dysfunction and cell death (enhanced ROS signaling, metabolic reprogramming, and release of mitochondrial DNA/ROS acting as danger molecules, ultimately leading to cell death). Lastly, as a result of incomplete repair or severe damage, TECs can undergo transient cell cycle arrest, as a protective mechanism to ensure genome stability. However, if persistent, this leads to a pro-inflammatory and profibrotic secretome, ultimately leading to fibrosis.

**Figure 2:**
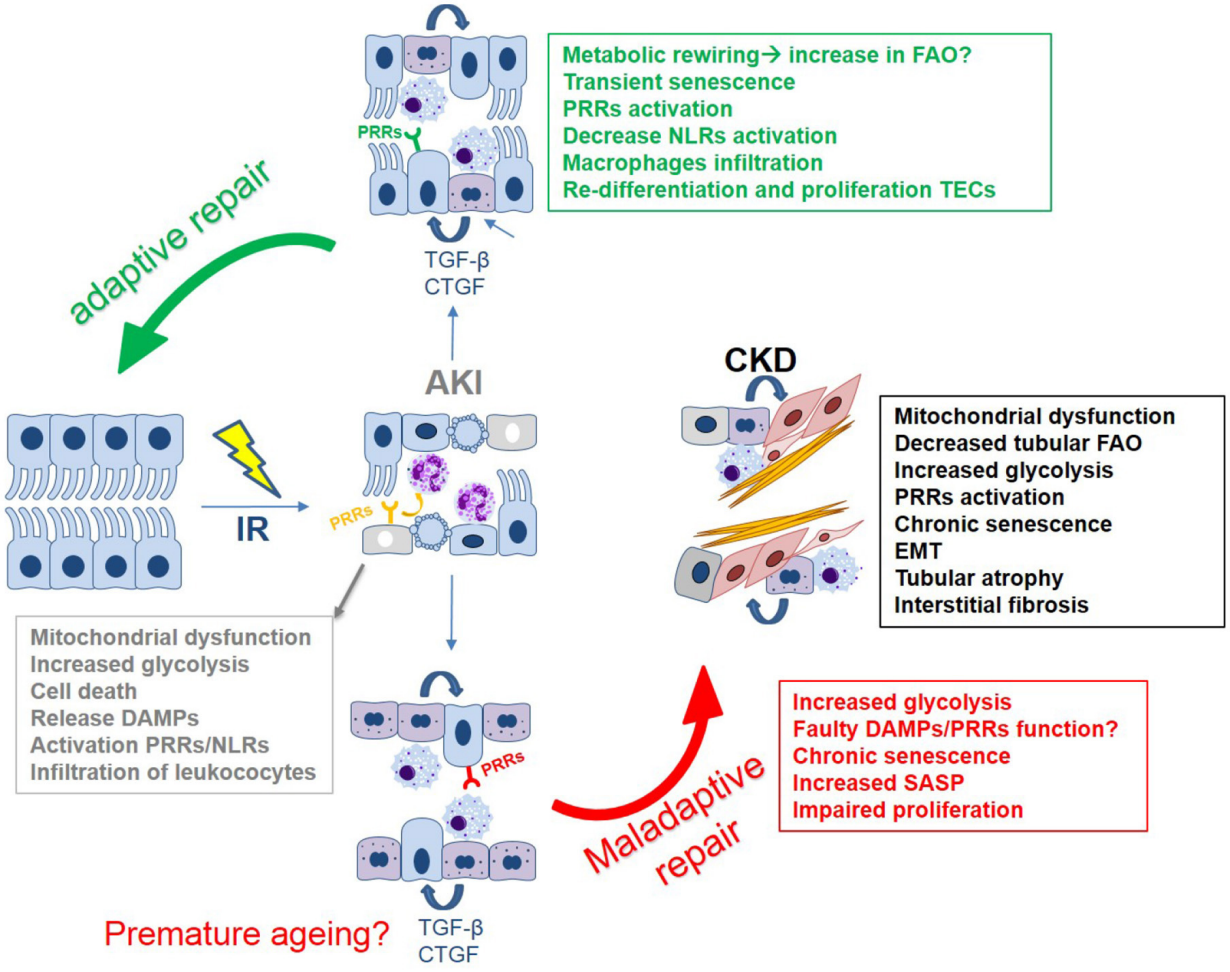
The tubular epithelial response to IR: a simplified overview of the main mechanisms driving (mal)adaptive repair leading to kidney fibrosis. Key mechanisms include: epithelial innate immune response, cell death, mitochondrial dysfunction, metabolic reprogramming, and cell cycle arrest/cellular senescence.

It has been well-established that mitochondrial dysfunction is a principal mediator of AKI through decreased ATP production, oxidative stress, mitochondrial DNA (mtDNA) release, and cell death. Mitochondria also play a crucial role in maintaining organelle function in cells. Organelle stress and crosstalk in the AKI-to-CKD transition has been recently reviewed (64). The ROS generated upon re-oxygenation and the inability to maintain endogenous antioxidant levels, results in mitochondrial oxidative stress and promotes AKI (88). Enhancement of the antioxidant defense via mitochondria-targeted approaches has been successful in ameliorating IRI-induced AKI. These have been summarized in Table 1. Additionally, a visual representation of the phenotypic changes occurring in TECs after IRI and the majority of the mechanisms described in this chapter can be found in Figures 1, 2.

**Table 1:** Mitochondria-targeted approaches in renal IRI.

**Experimental model**	**Species**	**Compound/approach**	**Function**	**Findings**
Unilateral IR, 30 min clamping	Rat	**Fasting** for 3 days before IRI	Fasting promotes mitochondrial biogenesis through the AMPK pathway	Decrease kidney damage, oxidative stress, and ameliorates mitochondrial function and has long-term beneficial effects (89).
Bilateral IR, 45 min clamping	mouse	**MitoQ** 4 mg/kg injected into the tail vein 15 min before ischemia	Mitochondria- targeted antioxidants	Decreases oxidative damage and improves renal function (90).
Unilateral IR, 40 min clamping	Rat	**SkQR1** 100 nmol/kg injected ip 3 h before IR, 1 h after IR, and subsequent injections at 13, 25, and 37 h; for a total of 500 nmol/kg.	Mitochondria- targeted antioxidants	Decreases oxidative damage and improves renal function (91).
Bilateral IR, 30–45 min clamping	rat	**SS-31** (0.5, 2.0, or 5.0 mg/kg) injected 30 min before IR, at the onset of reperfusion and 2 h later.	Mitochondria-targeted antioxidants	Decreases mitochondrial swelling, improves mitochondrial pathology and protect cristae membranes. Protects rats from kidney dysfunction, inflammation, and fibrosis (92, 93).
Unilateral IR, 15 min clamping	rat	Bolus injection of **glycine** before induction of renal IR but also a diet containing 5% glycine postoperatively for 2 weeks.	Amino acid with an important role in metabolic regulation and anti-oxidative reactions.	Improved renal function, decreased tubular injury, and oxidative stress. Prevents initial damage and chronic hypoxia (94).
Unilateral IR, 60 min clamping	Rat	**Resveratrol** (0.23 μg/kg body weight) via intragastrical administration (1.5 mL) 30 min before IR	Antioxidant	Decreased renal dysfunction, oxidative stress and inflammation. Ameliorated renal histologic damage (95).

### Senescence-Mediated (Mal)adaptive Repair

Kidney regeneration after IRI is accomplished by active tissue repair, in which the role of macrophage phenotype is crucial, as described in recent work (96). A balanced inflammatory response, together with a moderate level of damage, establishes an optimal microenvironment in which surviving TECs fully repair (3, 97). In this scenario of adaptive repair, the kidney regenerates over the course of a few days without scarring. The progression of AKI-to-CKD is the result of maladaptive tubular repair, which can be mediated by TEC senescence.

The hypoxic injury activates a DNA damage response (DDR) in TECs [Ataxia-Telangiectasia, Mutated(ATM)/ATM and Rad3-related(ATR) pathway] which results in cell cycle arrest (71, 98–100). This allows TECs to repair the damage and avoid further amplification of the vicious cycle of injury-induced cell death. ATR, an enzyme involved in DDR activation has been shown to protect against maladaptive tubular repair (100), further demonstrating a crucial role for DDR activation in TEC repair.

One of the consequences of irreparable DNA damage and mitochondrial dysfunction, especially in cells with a high energy demand, is a proliferation arrest termed “senescence” (101). Senescence is generally regarded as an irreversible event (102); however, there are reports that describe senescent cells being able to re-enter the cell cycle, suggesting that more research is needed to understand which scenario determines the fate of these cells (103). Senescent TECs, identified by different markers, with Senescence-associated beta-galactosidase (SA-β-gal) and the anti-proliferative proteins p16 and p21 being the most common, seem to accumulate acutely following experimental and human IRI (53, 104), as a consequence of oxidative stress, the so-called stress-induced senescence. *In vivo* studies using the renal IRI model showed that elimination of these cells might hinder regeneration (104, 105). Indeed, senescence also plays a key role in the healing of wounds, tissue repair, and during embryonic development (106). Acute and transient senescence, where damaged cells are eliminated, clearly has beneficial effects for an organism and in the adaptive tubular repair after IRI. Paradoxically, subtle accumulation of senescent cells (chronic senescence), instead, impairs the kidney's regenerative capacity, leading to AKI-to-CKD transition (52, 107, 108).

In this scenario, the use of senolytics (aimed at clearing senescent cells) seems a promising therapeutic strategy (49). However, given the non-specificity of current senescence markers and the existence of different senescence programs, elegantly reviewed by the De Maria group, more research is needed for this novel potential therapeutic field (109). The intervention strategies aimed at senescent cell removal or modulation, which seem to be effective in limiting the progression of IRI, have been reviewed previously (104). Nonetheless, having a specific biomarker for tubular senescence burden could be an effective way to stratify patients that could benefit from the use of senolytics.

Despite being arrested in the cell cycle, senescent cells remain metabolically very active and become fibrogenic due to their innate ability to create a pro-inflammatory environment and secrete pro-fibrotic molecules, components of the senescence-associated secretory phenotype (SASP) (110). Activation of SASP in TECs leads to activation and proliferation of fibroblasts and perivascular pericytes, which in turn induce extracellular matrix production and tubulointerstitial inflammation, with impaired M2 macrophage conversion (47, 71, 96). Perhaps redirecting the metabolism of senescent cells could be an effective way to turn their detrimental phenotype and fate. Recent studies have indeed highlighted the plasticity of these cells, being able to re-enter the cell cycle, posing a new challenge to the postulated theory, that cells can instead be re-programmed to change their cell fate (111).

In addition to the common components of the SASP, senescent cells produce exacerbated levels of mitochondrial-derived ROS and might also release mtDNA, which can act as a DAMP, further fueling PRR activation (101, 112). Collectively, they amplify the vicious cycle of inflammation, mitochondrial dysfunction, and senescence, ultimately leading to maladaptive tubular repair. Our group has recently described that the innate immune receptor TREM-1 links mitochondrial dysfunction, tubular senescence and maladaptive repair after AKI. This is the first study linking epithelial immunometabolism to cellular senescence in the context of IRI (52).

### Epithelial Immunometabolism

TECs possess an incredible plasticity. By tightly controlling their metabolism, they are able to anticipate and adapt to constantly changing environments in both health and disease. Activation of the innate immune system and metabolic reprogramming are tightly linked (113). The theory of immunometabolism has been increasingly appreciated to drive effector functions in immune cells. Despite TECs being regarded as innate immune cells of the kidney, research on epithelial immunometabolism is still in its infancy, particularly in the context of renal IRI (113). In the last 2 years, our group has found that the metabolic choice of TECs is driven by either membrane-bound or intracellular receptors of the innate immune system, proposing the involvement of epithelial immunometabolism in the pathogenesis of IRI and its detrimental consequences (51, 52).

The effect of renal injury on cellular metabolism has been well-established in various AKI models. The induction of AKI with mercuric chloride results in increased glycolytic activity (114), while FAO has been reported to be reduced in folic acid nephropathy (72). The general consensus is that such metabolic shifts are likely necessary to facilitate the initial recovery process; however, their effects may become deleterious when the altered metabolic state persists. Specifically, numerous studies have reported the damaging effects of increased glycolytic activity in TEC repair following IRI. Increased levels of glycolysis and glycolytic enzymes have been observed in TECs that failed to re-differentiate and underwent atrophy (68). As mentioned earlier, Zhou et al. reported the renoprotective effect of PKM2 inhibition by the S-nitroso-CoA reductase system following bilateral IRI. This obstruction of the glycolytic pathway was accompanied by a shift toward the pentose phosphate pathway (PPP), which generates Nicotinamide adenine dinucleotide phosphate (NADPH) and aids in the supplementation of antioxidant reservoirs, thereby offering protection against ROS-induced injury (87). Similarly, Kim et al. report the renoprotective effect of TP53 Induced Glycolysis Regulatory Phosphatase (TIGAR) activation in IRI, which leads to a metabolic shift away from glycolysis and employs the redox protection of the PPP (115). These studies emphasize the need for 'metabolic fluidity' while simultaneously displaying the need for strict metabolic control during IRI. Results from our group and that of others have shown the effect of TEC-specific expression of PRRs during IRI (8), while our recent reports demonstrate the direct involvement of innate immune signaling (via NLRX1 and TREM1) in regulating TEC metabolism following IRI (51, 52). Altogether these data show the inextricable connection between innate immune signaling and cellular metabolism in TECs, which ultimately defines their fate following an acute insult. It is for these reasons that immunometabolism should be recognized as a crucial mechanism through which TECs respond to, and recover from, IRI.

## PRR_s_, the Inflammasome and Their Ligands in Experimental AKI

### Toll-Like Receptors

In addition to their function as gatekeeper, protecting against micro-organism invasion, TLRs can be activated by a wide range of DAMPs released upon tissue injury. Upon IRI, tubular necrosis occurs together with the release of potential DAMPs, including High mobility group box 1 (HMGB1), histones, heat shock proteins, S100A8/A9, hyaluronic acid, biglycan, mtDNA, ROS, and ATP, which can function as ligands for PRRs. The type of ligands recognized by a specific TLR is partially dependent on their intracellular localization. One group can be found on the cellular surface and includes TLR2 and TLR4 and the other group, including TLR3, TLR7, TLR8, and TLR9, is expressed in intracellular vesicles such as endosomes, lysosomes, and the endoplasmic reticulum. Upon ligand recognition, the intracellular TIR-domain of a TLR functions as a scaffold for the recruitment of specific adaptor proteins via homotypic interactions between their TIR-domains.

Signaling pathways activated downstream of these adaptor molecules promote the expression of pro-inflammatory cytokines, chemokines, and type I and type III IFNs. Although TLRs provide protection against a wide variety of pathogens, inappropriate or unregulated activation of TLR signaling can lead to chronic inflammatory and autoimmune disorders (116, 117).

#### TLR2/4

*Tlr2* and *Tlr4* mRNA is constitutively expressed by TECs and is upregulated upon IRI in mice (118). Both *Tlr2* and *Tlr4* overexpression in TECs after IRI induced an exaggerated inflammatory response, resulting in impaired renal function (8, 57, 119). Antisense oligonucleotides targeting *Tlr2* protected renal tissue and function after IRI in mice. Compared to the single KO mice, double *Tlr2* and *Tlr4* KO animals showed no additional protective effects (120). Likewise, mice deficient for *Myd88* or *Trif* were not significantly protected against IRI compared to WT animals (119). This might be the consequence of adaptive mechanisms in these genetically modified animals.

Inhibition of TLRs must take place in the acute phase of injury and cannot be longstanding. Indeed, it has been shown that TLR4 blockade during the recovery phase after IRI slows down the process of tubular repair after IRI in rodents (58). In light of these data and in the context of our review, we believe that inhibiting the endogenous ligands, rather than the receptor itself, might be a safer approach.

#### TLR2/4 Ligands

One such potential ligand is HMGB1. HMGB1 is a nuclear factor that is highly and ubiquitously expressed in nearly all cell types. Upon renal IRI, HMGB1 is overexpressed, and secondary to cell injury, HMGB1 can be leaked into the circulation and activate TLR2, TLR4, and TLR9 (see below). We and others showed that treatment with anti-HMGB1 antibodies reduced renal injury, inflammation, and dysfunction in a murine model of IRI (121, 122). This protective effect of anti-HMGB1 treatment was confirmed in a model of cold and warm renal IRI in miniature swine (123). Another potential agonist for TLR4 is uromodulin. Uromodulin, or Tamm-Horsfall protein, is a highly glycosylated protein, normally secreted by epithelial cells of the thick ascending limb of Henle's loop in the intraluminal compartment. The function of uromodulin remains elusive, but data suggest that uromodulin might prevent the formation of kidney stones and urinary tract infection (124). *In vitro*, uromodulin has been shown to activate TLR4 signaling in myeloid DCs and bone marrow-derived macrophages (125). Upon kidney injury, uromodulin can leak into the renal interstitial space, potentially leading to the activation of intrarenal resident macrophages and DCs. In contrast with our expectations, uromodulin-deficient mice have markedly fewer resident macrophages. Upon IRI, these uromodulin-deficient mice exhibit aggravated renal injury and impaired polarization of macrophages toward an M2 healing phenotype (126). The discrepancy between *in vivo* and *in vitro* data might eventually be due to the different forms of uromodulin, i.e., full-length or truncated, monomeric or aggregating form.

Histones released from dying cells were also found to exacerbate renal tissue injury in AKI in a TLR2/TLR4 dependent manner, and administration of anti-histone antibody suppressed renal inflammation and injury and improved renal function (127).

Among other DAMPs, the calcium binding proteins (S100A8/A9), ligands of TLR4 and the RAGE receptor, are released following renal IRI and seem to play a pivotal role in orchestrating the repair response after hypoxic damage. During the initial injury phase, immune cells, particularly macrophages, are polarized toward an M1-like phenotype and produce pro-inflammatory cytokines, such as TNF-alpha and IL-6, which exacerbate inflammation. However, in a later phase, they switch their phenotype to a more reparative one, the M2-like phenotype, which secretes anti-inflammatory factors, determining the resolution of inflammation and stimulating tubular regeneration (128). Our group showed that S100A8/A9 proteins play a role in this process of macrophage polarization. By controlling excessive M2 polarization, S100A8/A9 fine-tunes the adaptive response of the kidney to IRI-induced AKI (56).

#### TLR3

TLR3 is activated upon the binding of single or double-stranded RNA and induces an antiviral immune response, characterized by the production of type I IFNs. TLR3 is constitutively expressed in mouse and human TECs and is activated earlier (few minutes) than TLR2 and TLR4 after IRI. In a model of bilateral IRI, *Tlr3*-deficient mice were significantly protected against kidney injury as indicated by less inflammation, less tubular apoptosis and necrosis, and preserved renal function 24 h after reperfusion (129). How TLR3 is involved in IRI remains speculative. Since *Tlr3* KO mice had diminished TRIF protein expression, reduction of necroptosis might be one explanation. Also, type 1 IFN-mediated auto- and paracrine activation of IFN receptors might contribute, for instance, to pyroptosis via transactivation of caspase-11 (130, 131). The effects of TLR3 in the later phases of reperfusion were not studied in this paper.

#### TLR3 Ligands

Double-stranded RNA released upon viral replication is the major ligand of TLR3, but TLR3 can also recognize mRNA and mRNA-protein complexes released by necrotic cells (132). Therefore, it is tempting to speculate that both mRNA and mRNA-protein complexes might amplify the pro-inflammatory loop via their interaction with TLR3 upon renal IRI. However, scientific evidences are still lacking.

#### TLR9

TLR9 is a cytosolic DNA sensing receptor and has evolved to detect unmethylated CpG DNA, commonly found in microbial DNA and DNA viruses, and initiate the production of type I IFN and proinflammatory cytokines. TLR9 is highly expressed in professional innate immune cells, such as plasmacytoid DCs and macrophages but also in the kidney. In a model of moderate renal IRI, TLR9 was not involved in renal dysfunction (133). In contrast, in a model of severe IRI, we showed that *Tlr9* deficiency resulted in improved survival in mice but not in the improvement of renal function and kidney damage (134). Improved survival was associated with reduced plasma mtDNA content and a subsequent decrease in hepatic injury. Surprisingly, and somewhat in contrast with our study, a recent study by Han et al. showed that selective intestinal TLR9 deficiency led to increased ischemic AKI and was associated with remote intestinal and hepatic injury. Intestinal *Tlr9* deficiency was associated with enlarged Paneth cell granules and increased IL-17A expression (135). We would expect that *Tlr9* KO animals would also present with this phenotype, but caution must be applied when interpreting scientific results generated by genetically-modified animals.

The mitochondrial dysfunction, cellular stress, and cell death involved in renal IRI result in the liberation of mtDNA, both in murine models of IRI and after renal transplantation in humans. Recently, our group reported a correlation between urinary mtDNA levels and the occurrence of DGF following renal transplantation (136). Outside of the mitochondrial matrix, mtDNA acts as a DAMP that can elicit neutrophil-mediated injury through TLR9, and other receptors (137). Indeed, the cyclic GMP–AMP synthase (cGAS)-stimulator of interferon genes (STING) pathway is also able to recognize cytosolic DNA. Cytosolic cGAS binds double-stranded DNA and catalyzes the production of the novel second messenger 2′-3′-cyclic AMP-GMP (2′3′-cGAMP) from ATP and GTP. The binding of cGAMP to the ER-resident protein STING releases DNA-triggered signals and activates the innate immune system (138, 139). Recently, Maekawa et al. showed that cytosolic translocation of mtDNA leads to tubular inflammation via the cGAS-STING pathway, linking mitochondrial dysfunction to enhanced inflammation (112). cGAS-STING is activated in the cortex of animals 24 h after renal IRI and *Sting* KO animals display mild tubular injury and inflammation. Therefore, we cannot exclude the possibility that the absence of reprotection in *Tlr9* KO animals, described in (134) after renal IRI, can be explained by an additional contribution of cGAS-STING. As cGAS-STING is activated in other pathogenic processes such as renal fibrosis (140) and senescence (141), further study into its effect on tubular repair will shed more light on the role of immunometabolism in AKI-to-CKD progression. Altogether, these studies strongly suggest that mitochondrial protection and a decrease in oxidative stress can be an elegant way to prevent AKI.

#### TREM-1

Triggering receptor expressed on myeloid cells-1 (TREM-1) is an activating receptor located primarily on cells of the innate immune system and some parenchymal cells (142). TREM-1 signals through its adapter protein, DNAX-activating protein (DAP12), to activate transcription factors capable of inducing the expression of pro-inflammatory cytokines and chemokines. TREM-1 can initiate inflammation, but can also work synergistically with TLRs to enhance an inflammatory response. Although initially studied in the context of infectious diseases, TREM-1 is also active in sterile inflammation (142). TREM-1 seems to be a hypoxia-inducible gene in myeloid DCs and TECs (52, 143) and might be involved in regulated cell death through amplification of inflammatory signals leading to necroptosis and pyroptosis, as has been shown in brain microglia (144). Interestingly, however, TREM-1 was also shown to mediate an inhibitory effect on necroptosis and pyroptosis in neonatal lung tissue, as suggested in previous work by Syed et al. (145). In our hands, in the early phase of renal IRI, TREM-1 modulation did not affect tubular damage or renal function (146). However, we did find that mice lacking TREM-1 displayed maladaptive repair characterized by persistent tubular damage, inflammation, fibrosis, and mitochondrial dysfunction-induced TEC senescence when exposed to renal IRI (52).

### Inflammasomes

#### NOD1/2

Nucleotide-binding oligomerization domain (NOD) 1 and 2 are part of the NOD-like receptor (NLR) family of cytoplasmic PRRs. NOD1/2 can either initiate immune responses to pathogenic invasion through the recognition of PAMPs or sterile inflammation in response to DAMPs released upon cell stress (8). Both NOD1 and NOD2 contain 3 basic structural units: a NOD region, a caspase recruitment domain (CARD), and a ligand-binding domain. Once activated through ligand binding, the resulting oligomerization leads to the binding of signaling molecules to CARD. NOD1/2 activation can lead to apoptotic signaling through RIP-like interacting caspase-like apoptosis-regulatory protein kinase (RIP2) or the release of inflammatory chemokines and cytokines (147). NOD1/2 are expressed on TECs in both mice and humans (148), and due to their structural resemblance to other NLRs and TLRs that are known to play a role in kidney disease, it is plausible that NOD1/2 may play a role in renal injury. Indeed, a study by Shigeoka et al. found that *Nod1/2* double KO led to a reduction in apoptosis following IRI (148). Expression of the pro-inflammatory cytokines IL-6, KC, and TNF-alpha were also reduced in mice deficient for NOD1/2, leading to the general suppression of the inflammatory response to IRI. Interestingly, NOD2-deficient mice were better protected against renal injury than NOD1-deficient mice, indicating that although both NOD proteins are involved in renal injury, the underlying mechanisms may be different (148). Endogenous ligands for NOD1/2 remain unknown, therefore, their discovery would certainly aid in the development of a therapeutic intervention.

#### NLRP3

NLRP3 (NOD-, LRR-, and pyrin domain-containing 3) is by far the best-characterized inflammasome-forming protein in the kidney. Once activated, the cytosolic innate immune receptor NLRP3 initiates the assembly of an inflammasome, leading to an inflammatory form of cell death (pyroptosis) and the proteolytic activation of the IL-1beta family of pro-inflammatory cytokines. The NLRP3 inflammasome can trigger inflammation by sensing a wide range of stimuli, but the specific mechanisms are still unclear. Among other factors, K^+^ efflux, ATP released from damaged mitochondria, and ROS production promote NLRP3 inflammasome activation (149).

*Nlrp3* gene expression in murine kidneys increased after IRI and peaked 5 days after reperfusion, corresponding to the repair phase. Although the *Nlrp3* gene was primarily expressed by leukocytes, TECs also expressed *Nlrp3* after hypoxia or LPS stimulation (150). In a murine model of bilateral IRI, we showed that *Nlrp3*-deficient animals were protected against mortality, renal dysfunction, and displayed a reduced influx of neutrophils into the kidneys despite similar degrees of tubular necrosis. In this study, activation of NLRP3 was triggered, in part, through ATP produced by mitochondria released from necrotic cells (151).

Uromodulin has also been shown to activate the NLRP3 inflammasome in human peripheral blood mononuclear cells, leading to the secretion of IL-1beta. However, as previously mentioned, uromodulin-deficient mice displayed aggravated renal injury upon IRI. This illustrates the complex role of uromodulin in regulating inflammation.

The role of leukocyte- vs. renal-associated *Nlrp3* expression has been studied in chimeric mice. In the early phase following IRI (day 1) only renal *Nlrp3* contributed to renal dysfunction based on serum creatinine. In contrast, 5 days after reperfusion (repair phase) both renal- and leukocyte-associated *Nlrp3* mediated loss of renal function. Interestingly, *Nlrp3*-deficient TECs showed increased proliferation and a superior repair response both *in vivo* and *in vitro* when compared to wildtype TECs (150).

More recently, it was shown that NLRP3 relocalizes from the cytosol to the mitochondria in TECs during hypoxia. The deletion of NLRP3 in TECs resulted in less mitochondrial ROS production, less mitochondrial damage, and less apoptosis in a model of *in vitro* hypoxia (152). Understanding the mechanisms of NLRP3 inflammasome activation will boost the development of small-molecule inhibitors for the treatment of NLRP3-related diseases (153).

#### NLRC5

NLR family CARD domain containing 5 (NLRC5) protein is a recently identified member of the NLR family that interferes with the assembly and activity of the NALP3 inflammasome complex by competing with ASC for pro-caspase-1 binding. Although NLRC5 activity leads to caspase-1 activation, induction of pyroptotic cell death dependent on NLRC5 has not yet been shown (154). *Nlrc5* is significantly upregulated in the kidney 24 and 48 h after IRI. *Nlrc5* deficiency significantly ameliorated renal function, injury, and inflammation 24 and 48 h after IRI. This was associated with less apoptosis in TECs and reduced inflammation in the kidneys (155).

#### NLRX1

In contrast to the hereinabove studied innate immune receptors, NLR family member X1 (NLRX1) exerts inflammasome independent anti-inflammatory effects by interfering with the canonical NF-kB signaling via inhibition of TRAF6 binding to IkB kinase (156). A unique feature of NLRX1 is its localization in the mitochondria. In 2017, we reported that NLRX1 protects against mortality and renal dysfunction after IRI by preventing excessive oxidative stress. We found that NLRX1 may act as an inhibitor of mitochondrial activity and prevents excessive oxidative stress, thereby preventing apoptosis of TECs during IRI (51).

Contrary to the studies by Zhou and Kim, we discovered renoprotective effects of glycolysis in IRI through our studies of NLRX1 (87, 152). Genetic deletion of *Nlrx1* potentiates mitochondrial oxidative phosphorylation in TECs, while glycolysis results in enhanced oxidative stress after ischemia and ultimately increases cell death. Using a KO mouse model for acute renal IRI, we found that NLRX1 deficiency enhanced oxidative stress, thereby profoundly enhancing tubular apoptosis, renal dysfunction, and mortality, in the early days after IRI (51). However, the consequences of this metabolic rewiring toward glycolysis on epithelial repair have not yet been investigated. A proper investigation into our hypothesis on the renoprotective effects of NLRX1 in IRI would require NLRX1 activation. *In silico* studies have postulated polyunsaturated fatty acids as NLRX1 ligands, although further validation is required (157). As these are naturally occurring lipids, we propose that NLRX1 activation through nutritional supplementation may be a possible therapeutic approach to impede AKI-induced oxidative stress.

## Innate Immunity in Human IRI and AKI

### Pattern Recognition Receptor Expression and Variation During Human IRI

Little is known about the direct *in vivo* role for PRRs in human AKI and specific data on the spatiotemporal dynamics of activation are lacking. Instead, most of the studies conducted in humans that investigated PRRs tried to identify single nucleotide variants related to certain outcomes as a weak proxy for a human knock-out or knock-in model, depending on the (often estimated) consequence of the genetic variant. We previously conducted a comprehensive screening of TLR single nucleotide variants (*TLR1-8* + *SIGIRR*) that were of interest because of their estimated effect on protein function. In a cohort of over 1,000 matched donor and recipient DNA samples, neither donor (renal) nor recipient (inflammatory cells) genetic variants were associated with DGF and the calculated effect size was low for individual variants, even after stratification for deceased donor type or analyzing the effect of all *TLR* gene variants in bulk, as compared to a baseline prediction model of cold ischemia time, donor age, and recipient age (158). A smaller study from Germany was able to find a positive association between a *TLR3* gene variant and DGF, although for the other *TLR* genes they were unable to find a relation with DGF (159). Furthermore, in a Brazilian study by Nogueira et al., again, no association between several *TLR4* gene variants and DGF were found (160). In the same cohort, with a similar experimental setup, we were also unable to find *NLRP3* and *TREM1* gene variants associated with DGF (146, 161). These data altogether suggest that the potential impact of these PRR genetic variants do not determine whether a patient will develop acute tubular necrosis and DGF, even though they were estimated to result in a PRR protein anomaly. Redundancy among these relatively common PRR genetic variants might partly explain this lack of association, since complete knockout of the gene of interest is a very rare event and not covered in the variants tested. At the expression level, polymerase chain reaction on implantation (time = 0) renal transplant biopsies showed a higher expression of *TLR4* and *MYD88* mRNA in deceased vs. living donor kidneys, but the authors could not find an association with the development of DGF (162). In an interesting study by McGuinness and colleagues from Glasgow, multi-omics analysis on pre- and postperfusion biopies (RNA sequencing, DNA methylation by whole genome bisulphite sequencing, and western blotting) identified a panel of expression markers associated with the development of DGF. They identified a transcriptional panel for DGF that was associated with innate immune signaling, TREM1 signaling, PRRs, and B cell development, with top-ranked networks related to immune system activation, cell death, and survival and cellular fitness. It seemed that cellular stress and restoration of physiological homeostasis (i.e., regeneration) was exacerbated in patients who developed DGF. At the protein level, DGF and perfusion status were associated with a state of cellular senescence (163). Altogether, the data from this study put PRR signaling, cell death and survival signaling, cellular stress and fitness, an exacerbated regenerative response, and cellular senescence at the center stage in the development of human IRI, and the authors suggest that these events probably occur independently of (minor) genetic differences in PRR. These conceptual findings seem to be in line with animal models.

### The MABSOT Project and the Human TLR2 Opsona OPN-305 Trial

As mentioned before, within the context of the EU FP7 MABSOT project, the humanized anti-TLR2 monoclonal antibody (OPN-305) was tested for the treatment of DGF after transplantation. In 2016, a phase I/II multicenter, randomized, double-blind placebo-controlled trial (NCT01794663) (164) in selected individuals at high risk for DGF showed that a preferred dose of 0.5 mg/kg (lowest dose tested) resulted in a lower percentage of DGF (primary endpoint; 26.5% in the treatment arm vs. 29.4% in the placebo arm) and functional DGF (failure of serum creatinine to decrease at least 10% daily; 38.2 vs. 52.9%, respectively) (165). Doses of 1.5 and 5 mg/kg appeared to be associated with a higher percentage of functional DGF, possibly in part due to the low number of DGF cases in the placebo group, according to the writers of the preliminary report. Data on long-term follow-up of the enrolled patients is pending, and a follow-up randomized controlled trial specifically looking at the clinical impact of the 0.5 mg/kg in recipients of an extended criteria donor kidney is planned according to the preliminary report. What can we learn thus far from this efficacy MABSOT trial as a first proof-of-concept for PRR blockade during human IRI? An interesting finding from the trial is the efficacy of the lowest antibody dose tested. Mean duration of 100% TLR2 receptor occupancy for this dose was 1 day, whereas for 1.5 mg/kg this was 6 days, and for the highest dose this was even 13 days. The use of OPN-305 in the prevention of IRI is limited by the fact that it does not act specifically on TECs, and the systemic blocking of TLR2 in all cells might result in an increased risk for (severe) infections. It might be the case that TLR blockade in the reparative phase after IRI in humans results in delayed tubular regeneration and even maladaptive repair, increasing the risk of renal fibrosis and infections. Long-term follow-up, with respect to rejection rate and renal function, would be of great interest for further investigation. Efficacy came at the cost of serious infectious and renal side effects in a select number of patients, but these results are the first to suggest the therapeutic potential for early pharmaceutical manipulation of PRRs in the context of human renal IRI.

## Concluding Remarks and Future Outlook

**Figure 3:**
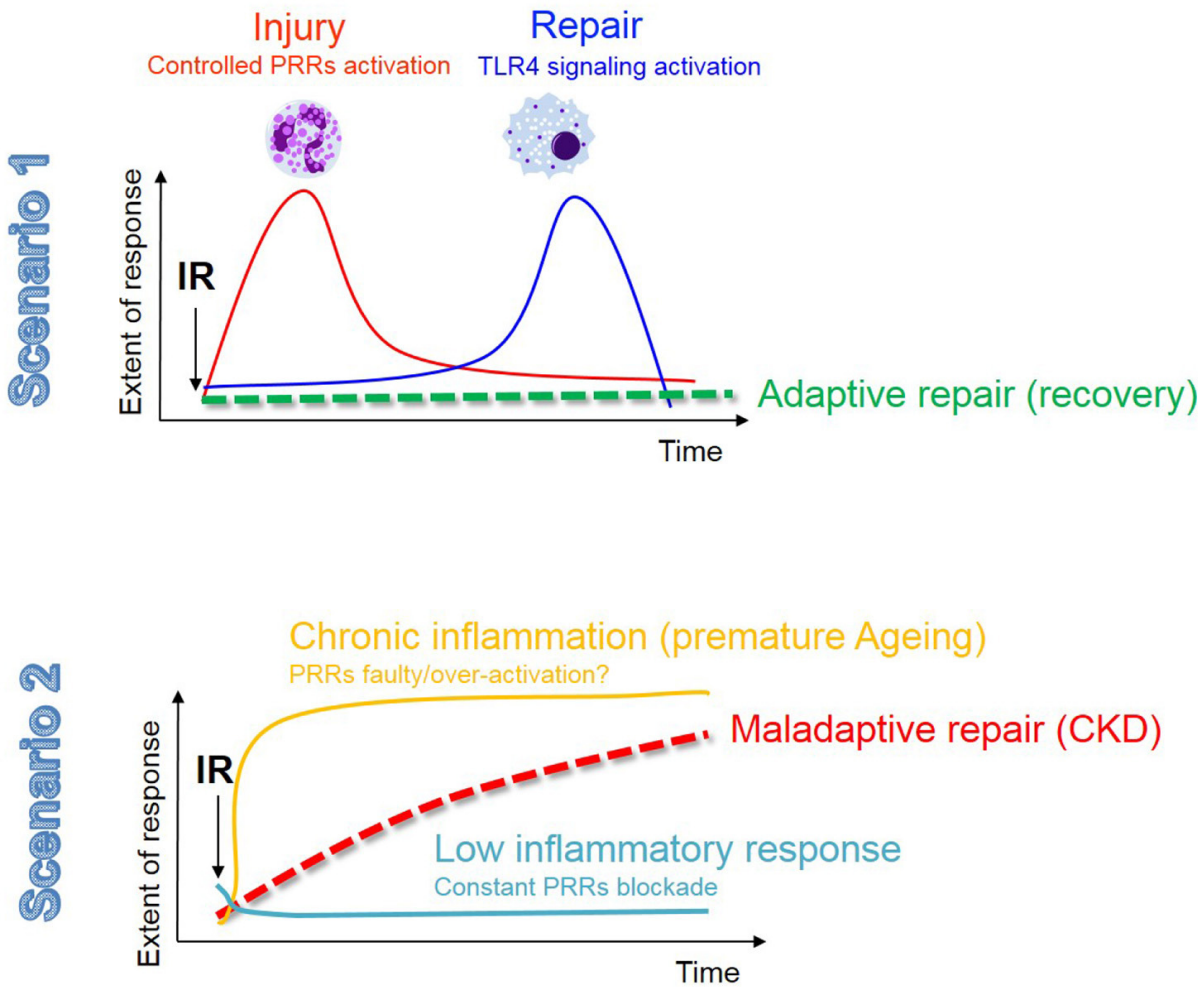
The innate immune response in renal IR: the fine balance between adaptive repair and chronic degeneration. In this figure we describe the 2 possible scenarios that may occur in the kidney following AKI. The fist scenario involves a moderate injury resolved by a balanced and timely activation of the innate immune response, most likely associated with a low and transient senescence burden, resulting in adaptive tubular repair and kidney regeneration. In the second scenario we envision that either a low-grade chronic inflammation (possibly related to an aging phenotype and the associated high senescent burden) or a constant PRRs blockade (to avoid excessive inflammation), contribute to phenotypic changes in TECs (as described in Figure 1) and maladaptive tubular repair.

This review described the main phenotypic changes occurring during renal IRI in TECs and the role of innate immunity in dictating inflammation and repair. The central dogma on the role of innate immunity in IRI still remains; excessive inflammation is just as detrimental as a faulty response, as both scenarios predispose to maladaptive repair and chronic progression (see Figure 3). We summarized the current evidence that the activation of innate immune receptors, in a very delicate balance (intensity, timing, cell types), is also necessary for adaptive repair. Moreover, here we have provided an additional and novel perspective about the role of epithelial metabolism and cell fate in IRI, which may be controlled by innate immunity.

As TECs have an incredible phenotypic plasticity and are among the most metabolically active cells, they are crucial for kidney function and homeostasis. Consequently, targeting epithelial immunometabolism holds great potential to alter the initiation of acute renal failure, but also progression towards CKD. As a final consideration, we are in a time where longevity is increasing and research on cellular senescence in the kidney, as a strategy to limit the AKI-to-CKD transition, is a fascinating area to explore for therapeutic potential. Unraveling differences between drivers of acute and chronic senescence will be essential to further investigate whether these cells could be trained to change their cell fate and function, in order to slow down the progression of kidney remodeling. Changes in mitochondrial homeostasis, metabolism, and function of innate immune receptors are known pathological mechanisms associated with aging, and thus could also take place in senescent TECs. Detailed studies on the hallmarks of senescent TECs in the different phases of renal IRI may shed light on novel mechanisms that can be targeted to redirect their phenotype and overcome the issue of repair by elimination. We envision that determining the therapeutic window of opportunity to regulate renal homeostasis by targeting innate immunity, immunometabolism, and cellular senescence should be among the future research goals in the field of nephrology and kidney transplantation.

## Author Contributions

AT, JK, and SF designed and directed the project. AS contributed to the study. All authors contributed to the article and approved the submitted version.

## Conflict of Interest

The authors declare that the research was conducted in the absence of any commercial or financial relationships that could be construed as a potential conflict of interest.
